# Epicardial adipose tissue volume is associated with impaired left atrial mechanics in hypertensive overweight/obese patients: the potential mediating role of insulin resistance

**DOI:** 10.3389/fnut.2026.1778193

**Published:** 2026-02-23

**Authors:** Shuang Yin, Xu Zhao, Qing Liu, Tingting Fu, Qiaobing Sun, Yu Pan, Mengxuan Wang, Bing Wang, Minghui Gong, Yan Lu, Chongfu Jia, Yinong Jiang, Yan Liu

**Affiliations:** Department of Cardiology, First Affiliated Hospital of Dalian Medical University, Dalian, Liaoning, China

**Keywords:** epicardial adipose tissue volume, hypertension, left atrial strain, overweight/obesity, triglyceride glucose-body mass index

## Abstract

**Background:**

Epicardial adipose tissue (EAT) is implicated in cardiovascular disease, but its specific impact on left atrial (LA) mechanics in hypertensive overweight/obese patients remains unclear. We investigated the association between EAT volume (EATV) and LA dysfunction in this high-risk population.

**Methods:**

In this cross-sectional study, 264 hypertensive overweight/obese patients (body mass index ≥24 kg/m^2^) underwent coronary computed tomography angiography (CCTA) for EATV quantification. Participants were stratified into low- and high-EATV groups by the median value (72.67 cm^3^). LA strain analysis, including reservoir strain (LAs-s), conduit strain (LAs-e), booster strain (LAs-a), and LA stiffness index (LASI), were assessed by two-dimensional speckle tracking echocardiography. The triglyceride glucose-body mass index (TyG-BMI) was calculated to reflect the insulin resistance. Multivariable regression and mediation analyses evaluated associations between EATV and LA mechanics.

**Results:**

The high-EATV group had significantly higher TyG-BMI and longer hypertension duration. Despite comparable conventional echocardiographic parameters, this group demonstrated impaired LA mechanics, evidenced by reduced LAs-s, LAs-e, and LAs-a, alongside elevated LASI. EATV was inversely correlated with LA strains (LAs-s, LAs-e, LAs-a) but positively correlated with both TyG-BMI and LASI. After multivariable adjustment, EATV remained independently associated with LAs-s, LAs-a, and LASI. Mediation analysis indicated that TyG-BMI explained 12.87% of the association between EATV and LAs-s, and 10.00% of the association between EATV and LASI.

**Conclusion:**

Increased EATV is independently associated with impaired LA mechanics in hypertensive overweight/obese patients. Insulin resistance, assessed by TyG-BMI, may partly explain these associations, linking epicardial adiposity to early atrial dysfunction.

## 1 Introduction

Obesity and hypertension represent intertwined global health crises, synergistically amplifying cardiovascular disease (CVD) risk. By 2030, 20% of adults (1.2 billion) worldwide are projected to have obesity (1). Concurrently, hypertension afflicts approximately 244 million adults in China alone, with a prevalence of 27.9%. Critically, obesity and hypertension frequently coexist, with over 60% of overweight/obese individuals having hypertension as a comorbidity, and obesity underlies 65–78% of essential hypertension. This synergy amplifies risks for heart failure (HF) and atrial fibrillation (AF) (2–4).

Epicardial adipose tissue (EAT), a key component of visceral fat accumulation, is deposited between the visceral pericardium and the myocardium, placing it in direct contact with the cardiac muscle. In obesity, EAT expansion not only promotes systemic inflammation, microcirculatory dysfunction, myocardial fibrosis, and adverse remodeling, but also exacerbates local and systemic insulin resistance (IR) (5). This aggravated IR, in turn, creates a vicious cycle that further impairs myocardial metabolism (6, 7). Excessive EAT deposition leads to mechanical inhibition and intramyocardial fat infiltration, correlating with subclinical cardiac dysfunction (8, 9), and heightens risks of coronary heart disease (CHD), AF, and heart failure with preserved ejection fraction (HFpEF) (10).

However, the impact of EAT on LA mechanics in overweight/obese patients with essential hypertension, and the potential mediating role of IR, remain unexplored. While the triglyceride-glucose (TyG) index is a recognized IR surrogate (11), the TyG-body mass index (TyG-BMI) has emerged as a novel marker that integrates adiposity for a more robust assessment of the metabolic burden of IR (12). Recent evidence also confirms TyG-BMI predicts IR more accurately than the TyG alone (13, 14). Coronary computed tomography angiography (CCTA), with high spatial resolution and reproducibility, is the preferred tool for EAT evaluation. Two-dimensional speckle tracking echocardiography (2D-STE) is a non-invasive, angle-independent method for assessing global and regional myocardial mechanics. Therefore, we employed CCTA and 2D-STE in this cross-sectional study to investigate the association between EATV and LA mechanics in hypertensive, overweight/obese patients, and to determine whether TyG-BMI-assessed IR mediates this relationship.

## 2 Methods

### 2.1 Study design and population

This cross-sectional observational study adhered to the Declaration of Helsinki and received approval from the Ethics Committee of the First Affiliated Hospital of Dalian Medical University (Approval No: PJ-KS-KY-2025-176). The ethics committee waived the requirement for written informed consent due to the retrospective nature of the study.

Between October 2021 and September 2024, we consecutively enrolled 264 overweight/obese adults (BMI ≥ 24 kg/m^2^) with essential hypertension, aged 18–75 years. Hypertension was defined according to 2024 hypertension Guidelines (15, 16). Overweight/obesity status was defined based on Chinese national criteria (BMI ≥ 24 kg/m^2^) (17). Exclusion criteria were as follows: secondary hypertension; coronary heart disease (prior angina pectoris, myocardial infarction, revascularization, or >50% coronary stenosis on CCTA); heart failure [left ventricular ejection fraction (LVEF) < 50%]; moderate or severe valvular disease; atrial fibrillation; severe hepatic or renal dysfunction [estimated glomerular filtration rate (eGFR) < 30 mL/min*1.73 m^2^]; malignancies; and inadequate echocardiographic image quality precluding reliable 2D speckle-tracking analysis. All participants underwent comprehensive assessment, including physical examination, laboratory tests, echocardiography, and CCTA.

### 2.2 Clinical and laboratory assessment

Demographic and hospitalization data were extracted from electronic medical records using standardized case-report forms. Key parameters included: anthropometrics (height, weight, blood pressure and heart rate); metabolic biomarkers fasting plasma glucose [FPG], glycated hemoglobin [HbA1c], total cholesterol [TC], triglyceride [TG], low-density lipoprotein cholesterol [LDL-C], high-density lipoprotein cholesterol [HDL-C], lipoprotein (a) [Lp(a)], apolipoprotein-A 1[Apo-A1], apolipoprotein-B [Apo-B], serum creatinine [Scr], uric acid [UA], and high-sensitivity C-reactive protein [hs-CRP]. The eGFR was calculated using the Chronic Kidney Disease Epidemiology Collaboration (CKD-EPI) formula (18). Additionally, we calculated the indices: the TyG using the formula: ln [TG (mg/dL) × FPG (mg/dL)/2], and the TyG-BMI as the product of TyG and BMI.

### 2.3 Echocardiography

All patients underwent echocardiographic evaluation using a commercially available system (Vivid E9, GE Vingmed Ultrasound, Horten, Norway) equipped with a 2.5–5.0 MHz phased-array transducer. Parameter measurements followed the guidelines of the American Society of Echocardiography (19). To ensure optimal image quality, particularly in overweight/obese patients, all examinations were conducted by experienced sonographers.

During examinations, patients were instructed to maintain calm breathing in the left lateral decubitus position. Electrocardiograms (ECGs) were recorded simultaneously, and two-dimensional ultrasound was used to measure parameters, including LV end-diastolic diameter (LVEDD), LV posterior wall thickness at end-diastole (LVPWTD), interventricular septum thickness at end-diastole (IVSTD), and LA diameter (LAD). LA maximal volume (LAV) was measured at end-systole using the biplane method of discs (modified Simpson’s rule) in apical 4- and 2-chamber views. LVEF was quantified via the same method. LV mass (LVM, g) was calculated using the Devereux formula: LVM = 0.8 × 1.04 × [(IVSTD + LVEDD + LVPWTD)^3^ − LVEDD^3^] + 0.6, and LVM index (LVMI, g/m^2^) = LVM/Body surface area (BSA). LAV index (LAVI) was calculated as LAV divided by BSA. Pulsed-wave Doppler was used to measure early (E) and late (A) mitral inflow velocities, and the E/A ratio was calculated. The deceleration time of early mitral inflow (EDT) was recorded. Tissue Doppler imaging was performed to obtain the myocardial velocity (e’) of the lateral mitral annulus in the apical 4-chamber view, and the E/e’ ratio was calculated.

**Figure 1:**
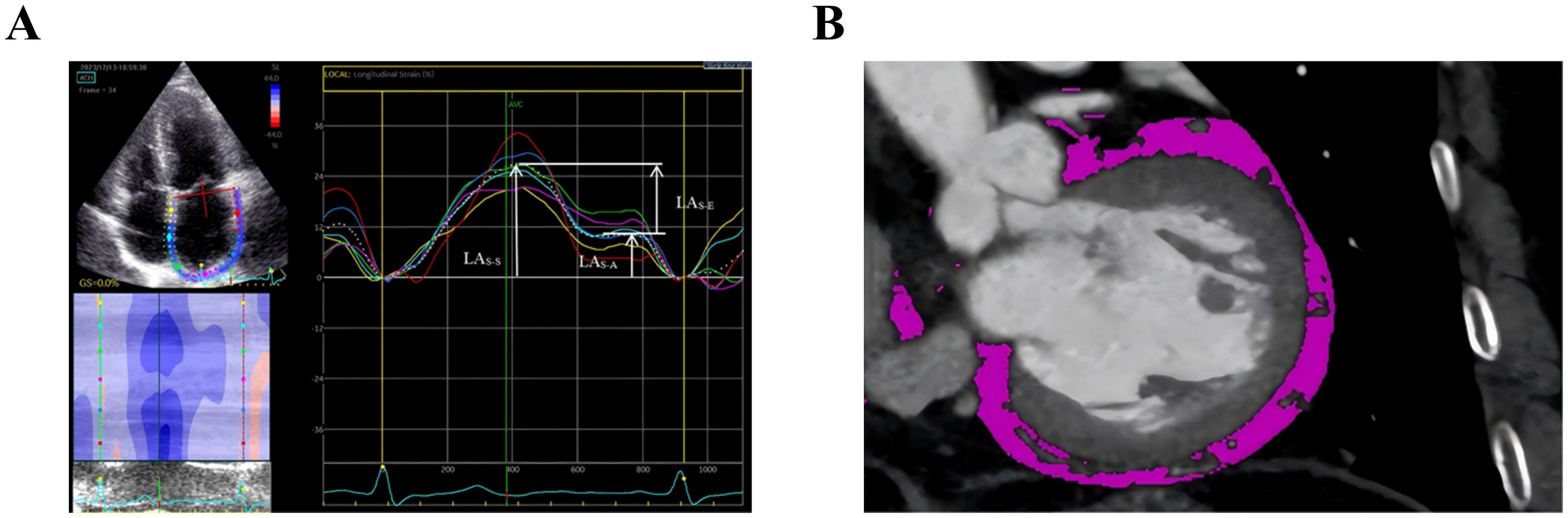
**(A)** Two-dimensional speckle tracking echocardiography (2D-STE) derived LA strain analyses from the apical four-chamber view. **(B)** EATV quantification assessed by coronary computed tomography angiography (CCTA).

Offline 2D-STE analysis was performed using EchoPAC software (version 202; GE Vingmed Ultrasound). Only echocardiographic loops with adequate image quality for accurate speckle-tracking analysis were included; loops with poor endocardial border definition were excluded. LA strain parameters, LA reservoir strain (LAs-s), LA conduit strain (LAs-e), and LA booster strain (LAs-a), were derived from apical 4- and 2-chamber views and average (20) (Figure 1A). The LA stiffness index (LASI) was defined as [E/e’]/LA_S-S_ (21).

### 2.4 EAT measurement

EAT quantification utilized dual-source CT (Somatom Definition Flash or Force, Siemens Healthineers, Germany). Prior to CCTA, patients received breath-hold training (>15 s) and antecubital intravenous access for iodinated contrast injection. All patients were administered 0.25 mg sublingual nitroglycerin (AstraZeneca) 5 min pre-scan to optimize coronary vasodilation. All patients maintained heart rate below 80 bpm during CCTA. Those exceeding this threshold received oral metoprolol (25–100 mg) 1 h pre-scan, with continuous blood pressure monitoring to detect hypotension. Patients were positioned supine with both arms elevated, and ECG gating was applied. Scanning extended from the tracheal carina to 1 cm below the diaphragm. Scout images optimized cardiac centering prior to acquisition.

Scanning parameters included: 250 ms rotation time; detector collimation 2 × 64 × 0.6 mm (Somatom Definition Flash) or 2 × 96 × 0.6 mm (Somatom Force); 66 ms temporal resolution; reconstructed slice thickness 0.75 mm with 0.7 mm increment. Tube setting (100–120 kV, 200–400 mAs) employed automatic dose modulation. Contrast administration followed a triphasic protocol: (1) 40–50 mL iodinated contrast (370 mgL/mL) at 5.5–6 mL/s. (2) 20–30 mL contrast at 3.5–4.0 mL/s. (3) 30 mL saline chaser at 4.0 mL/s.

CCTA images (Siemens Somatom Definition Flash/Force) were analyzed on a SyngoVia workstation (VB60, Siemens Healthineers, Germany). DICOM datasets underwent semi-automated segmentation of cardiac structures, followed by EAT quantification. EAT volume (EATV) and density were measured with a standardized attenuation threshold of −190 to −30 Hounsfield units (HU) (22) via the anatomical structure display module (Figure 1B).

### 2.5 Statistical analysis

Data normality was assessed using the Kolmogorov–Smirnov test. Normally distributed continuous variables are reported as mean ± standard deviation (SD), and non-normally distributed variables as median with interquartile range (IQR; 25th-75th percentiles). Categorical variables are presented as percentages (%). Between-group comparisons were performed using independent Student *t*-tests for normally distributed continuous variables, while Mann–Whitney *U* tests for non-normally distributed variables. The chi-square tests (*χ*^2^ tests) were applied for categorical variables. Correlation analysis employed Pearson’s correlation for normally distributed variables and Spearman’s rank correlation for non-normally distributed variables. Multivariable linear regression models were constructed to evaluate independent associations between EATV and LA strain parameters, with sequential adjustment for clinically relevant covariates. The results are presented as unstandardized regression coefficients (*β*), and the goodness-of-fit for each model was assessed using the adjusted *R*^2^ value. Model 1: sex, age, and BMI. Model 2: sex, age, duration of hypertension, history of diabetes mellitus (DM), smoking status, TyG-BMI, HDL, ApoA1, and eGFR. Model 3: Model 2 plus cardiometabolic medications [sodium-dependent glucose transporters 2 inhibitors (SGLT-2i), Glucagon-like peptide-1 receptor agonist (GLP-1RA), and statins]. Model 4: Model 3 plus conventional echocardiographic parameters LVEF and LVMI. Multicollinearity among independent variables was assessed using variance inflation factors (VIF). All VIF values were below 2, indicating no multicollinearity. Mediation analysis was conducted to assess the role of TyG-BMI in the relationship between EATV and LA mechanics. Given the cross-sectional design, mediation analysis was used to explore potential mechanistic pathways but does not establish causality. This was performed using Hayes’s PROCESS macro (version 4.0) in SPSS, with bias-corrected bootstrapping (5,000 resamples) to estimate 95% confidence intervals for indirect effects. All analyses were performed using SPSS (version 25.0; IBM Corp., Armonk NY). Figures were generated using Origin (2021; OriginLab Corp., Northampton, MA) and GraphPad Prism (version 9.5; GraphPad Software, San Diego, CA). A two-sided *p*-value <0.05 was considered statistically significant.

## 3 Results

### 3.1 Baseline characteristics

A total of 264 hypertensive patients with overweight or obesity were stratified into group I and Group II based on the median EATV. The baseline characteristics are detailed in Table 1. Group II had a significantly higher proportion of males, a higher BMI, and a greater prevalence of smokers compared to Group I. The duration of hypertension was also longer in Group II. Metabolic profiling revealed a higher TyG-BMI in Group II. This was accompanied by a more adverse lipid profile, characterized by lower HDL-C and reduced Apo-A1. The use of statins was significantly more frequent in Group II compared with Group I. No significant differences were observed between groups in terms of age, the prevalence of diabetes or obstructive sleep apnea-hypopnea syndrome, renal function, HbA1c, FPG, other lipid parameters, hs-CRP, BNP, or the use of antihypertensive and glucose-lowering medications.

**Table 1:** Comparison of clinical characteristics between different EATV groups.

Variables	Group I	Group II	*p*
	EATV < 72.67 cm^3^	EATV ≥ 72.67 cm^3^	
	*N* = 132	*N* = 132	
Demographic data			
Male, *n* (%)	73 (55.30)	99 (75.00)	0.001
Age, years	52 (41.00,61.00)	54 (45.00,63.75)	0.135
BMI (kg/m^2^)	28.02 (26.41, 30.37)	29.03 (27.10, 31.32)	0.035
Smoker, *n* (%)	43 (32.60)	61 (46.20)	0.023
Drinker, *n* (%)	26 (20.00)	34 (25.80)	0.267
Hypertension duration, years	5.00 (1.00, 10.00)	8.00 (3.00, 15.00)	0.003
Diabetes, *n* (%)	40 (30.30)	46 (34.80)	0.597
OSAS, *n* (%)	16 (12.10)	22 (16.70)	0.293
Medication history			
ARNI/ACEI/ARB, *n* (%)	72 (54.50)	81 (61.40)	0.262
β-blocker, *n* (%)	43 (32.60)	46 (34.80)	0.696
CCB, *n* (%)	93 (70.50)	94 (71.20)	0.892
SGLT-2i, *n* (%)	4 (3.00)	7 (5.30)	0.355
GLP-1RA, *n* (%)	2 (1.50)	1 (0.80)	0.561
Statins, *n* (%)	8 (6.10)	20 (15.20)	0.016
Laboratory data and biomakers			
HbA1c (%)	5.90 (5.60, 6.20)	5.90 (5.63, 6.48)	0.247
FPG (mmol/L)	5.06(4.63, 5.58)	5.18(4.75, 6.03)	0.171
TyG	8.65 (8.30, 9.07)	8.84 (8.42, 9.09)	0.076
TyG-BMI	246.20 (227.15, 272.21)	258.15 (234.26, 287.29)	0.015
TG (mmol/L)	1.51 (1.06, 2.01)	1.71(1.17, 2.31)	0.081
TC (mmol/L)	4.75 ± 0.92	4.81 ± 1.04	0.650
HDL-C (mmol/L)	1.08 (0.91, 1.26)	1.01 (0.86, 1.12)	0.029
ApoA1 (g/L)	0.96 (0.86, 1.09)	0.91 (0.84, 1.03)	0.043
ApoB (g/L)	0.87 (0.75, 0.99)	0.93 (0.75, 1.04)	0.294
LDL-C (mmol/L)	2.62 ± 0.63	2.71 ± 0.73	0.282
UA (μmol/L)	378.35 (310.13, 443.07)	373.85 (312.62, 450.32)	0.962
eGFR (mL/min/1.73 m^2^)	103.03 (92.77, 110.30)	104.79 (95.95, 111.00)	0.256
hs-CRP (mg/L)	1.25 (0.67, 2.90)	1.45 (0.77, 2.71)	0.483
Cys C (mg/L)	0.96 (0.84, 1.05)	0.95 (0.86, 1.10)	0.372
BNP (pg/mL)	18.92 (9.15, 32.00)	19.40 (7.70, 43.72)	0.562

Data are expressed as mean ± standard deviation, median (interquartile range) or number (percentage).

EATV, epicardial adipose tissue volume; BMI, body mass index; OSAS, obstructive sleep apnea–hypopnea syndrome; ARNI, angiotensin receptor neprilysin inhibitor; ACEI, angiotensin converting enzyme inhibitors; ARB, angiotensin receptor blocker; β-blocker, β-adrenergic receptor antagonist; CCB, calcium channel blocker; SGLT-2i, sodium-dependent glucose transporters 2 inhibitors; GLP-1RA, glucagon-like peptide-1 receptor agonist, Statins HMG-CoA reductase inhibitors; FPG, fasting plasma glucose; HbA1c, glycated hemoglobin; TyG, triglyceride glucose index; TyG-BMI, triglyceride glucose-body mass index; TG, triglyceride; TC, total cholesterol; HDL-C, high density lipoprotein cholesterol; LDL-C, low density lipoprotein cholesterol; ApoA1, Apolipoprotein A1; ApoB, Apolipoprotein B; UA, uric acid; eGFR, estimated glomerular filtration rate; hs-CRP, high-sensitivity c-reactive protein; Cys C, Cystatin C; BNP, B-type natriuretic peptide.

### 3.2 Echocardiographic parameters

**Figure 2:**
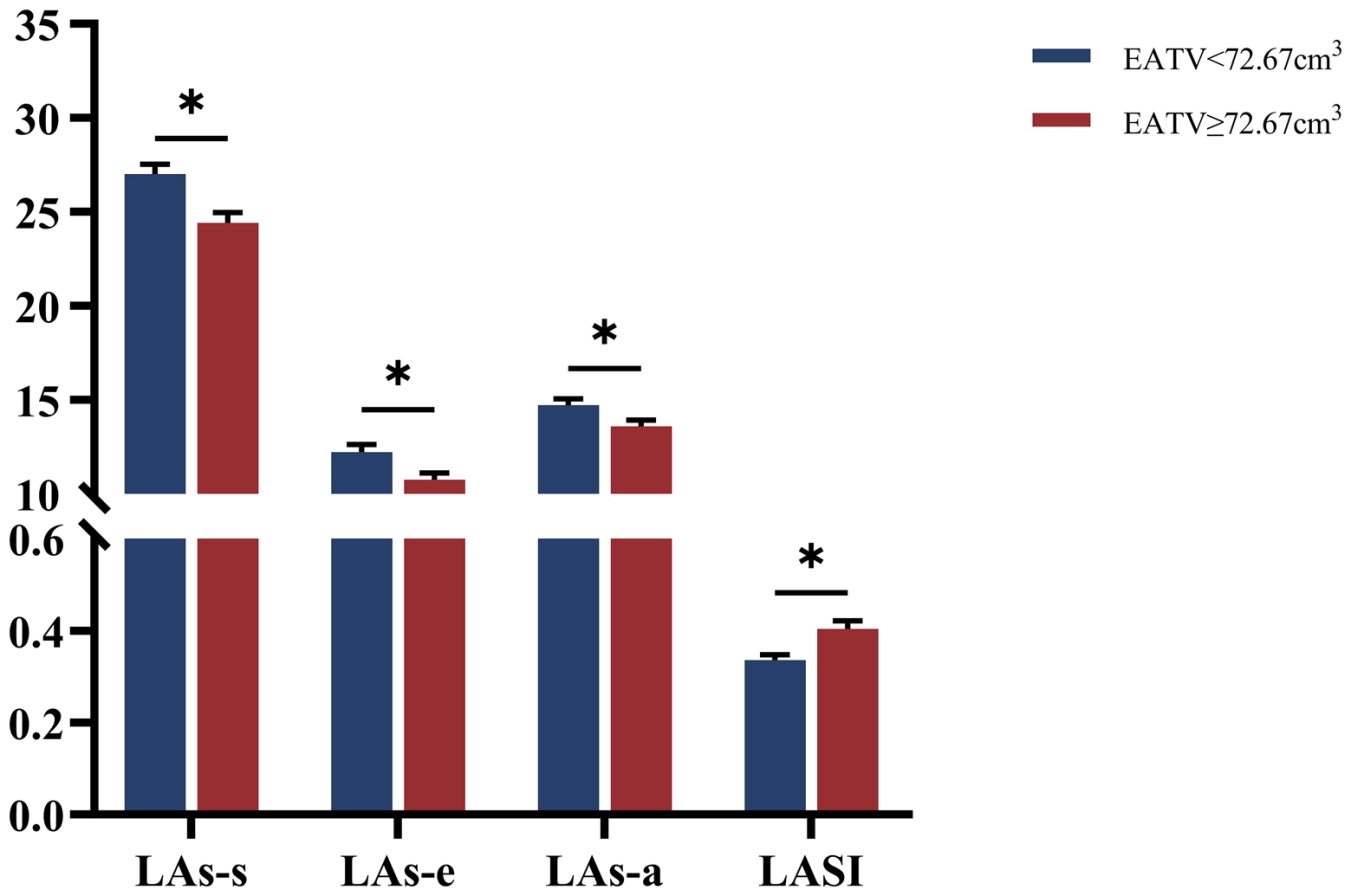
Comparison of left atrial mechanics between different EATV groups. Data are presented as mean ± SEM.

Conventional echocardiographic parameters, including LVEF, E/A ratio, E/e’ ratio, LAVI, and LVMI, did not differ significantly between the two groups (Table 2). However, a comprehensive assessment of LA mechanics using 2D-STE revealed significant impairments in Group II. Patients in the high-EATV group demonstrated significantly reduced reservoir strain, conduit strain, and booster pump strain. Furthermore, LASI was significantly elevated in Group II compared to Group I (Figure 2).

**Table 2:** Echocardiographic characteristics across different EATV groups.

Variables	Group I	Group II	*p*
	EATV < 72.67 cm^3^	EATV ≥ 72.67 cm^3^	
	*N* = 132	*N* = 132	
E/A (cm/s)	0.86 (0.74, 1.11)	0.84 (0.73, 1.07)	0.561
LVEF (%)	60.00 (58.25, 60.00)	60.00 (59.00, 60.00)	0.914
E/e’ ratio	8.36 (6.80, 10.00)	9.00 (7.00, 11.00)	0.061
LAVI (mL/m^2^)	29.24 (22.69, 36.16)	29.98 (24.40, 37.36)	0.347
LVMI (g/m^2^)	93.25 (82.01, 110.92)	99.97 (87.71, 115.07)	0.067
LAs-s (%)	27.00 ± 6.26	24.40 ± 6.44	0.001
LAs-e (%)	11.63 (8.98, 15.40)	10.00 (7.58, 13.48)	0.007
LAs-a (%)	14.34 (12.41, 16.95)	13.50 (11.02, 15.90)	0.024
LASI	0.31 (0.22, 0.40)	0.37 (0.28, 0.48)	0.001

EATV, epicardial adipose tissue volume; E/A, early to late diastolic transmitral flow velocity ratio; LVEF, left ventricular ejection fraction; e’, mitral annular early diastolic velocity; LAVI, left atrial volume index; LVMI, left ventricular mass index; LAs-s, left atrial reservoir strain; LAs-e, left atrial conduit strain; LAs-a, left atrial booster strain; LASI, left atrial stiffness index.

### 3.3 Correlation analysis

**Figure 3:**
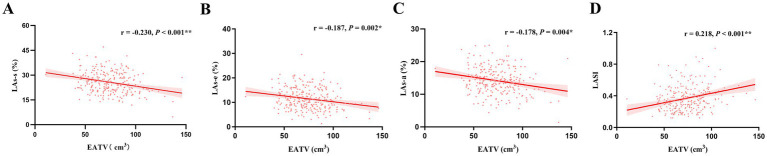
Correlation analysis of EATV with left atrial mechanics. **(A)** EATV and LAs-s. **(B)** EATV and LAs-e. **(C)** EATV and LAs-a. **(D)** EATV and LASI. EATV, epicardial adipose tissue volume; LAs-s, left atrial reservoir strain; LAs-e, left atrial conduit strain; LAs-a, left atrial booster strain; LASI, left atrial stiffness index.

**Figure 4:**
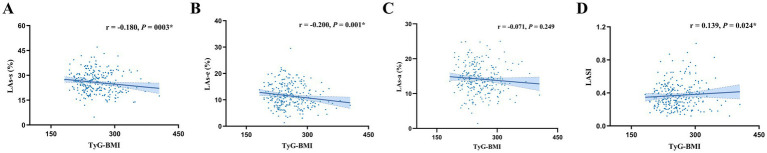
Correlation analysis of TyG-BMI with left atrial mechanics. **(A)** TyG-BMI and LAs-s. **(B)** TyG-BMI and LAs-e. **(C)** TyG-BMI and LAs-a. **(D)** TyG-BMI and LASI. TyG-BMI, triglyceride glucose-body mass index; LAs-s, left atrial reservoir strain; LAs-e, left atrial conduit strain; LAs-a, left atrial booster strain; LASI, left atrial stiffness index.

As detailed in Table 3, EATV demonstrated significant, albeit weak-to-moderate, positive correlations with several metrics of adiposity and insulin resistance, including BMI, FPG, TyG, TyG-BMI, and TG level. Conversely, EATV was negatively correlated with HDL-C. No significant correlations were observed between EATV and other lipid parameters, renal function markers, or inflammatory biomarkers. Crucially, EATV was significantly correlated with impaired LA function. It exhibited negative correlations with all LA strain parameters, including LAs-s, LAs-e, and LAs-a. Furthermore, a positive correlation was found between EATV and LASI. No significant correlation was observed between EATV and LAVI. Similarly, TyG-BMI showed significant negative correlations with LAs-s and LAs-e, as well as a positive correlation with LASI and LAVI. However, no significant correlation was observed between TyG-BMI and LAs-a (Table 4, Figures 3, 4).

**Table 3:** Correlation analysis of EATV with clinical indicators.

Variables	EATV (cm^3^)	
	*r*	*p*
BMI (kg/m^2^)	0.175	0.004
FPG (mmol/L)	0.132	0.037
TyG	0.154	0.012
TyG-BMI	0.210	0.001
TG (mmol/L)	0.141	0.022
TC (mmol/L)	0.052	0.404
HDL-C (mmol/L)	−0.130	0.035
LDL-C (mmol/L)	0.074	0.232
Apo-A1 (g/L)	−0.101	0.102
ApoB (g/L)	0.069	0.264
eGFR (mL/min/1.73 m^2^)	0.016	0.794
UA (μmol/L)	0.023	0.705
Cys C (mg/L)	0.086	0.163
hs-CRP (mg/L)	0.025	0.702
BNP (pg/mL)	−0.031	0.650

EATV, epicardial adipose tissue volume; BMI, body mass index; FPG, fasting plasma glucose; TyG, triglyceride glucose index; TyG-BMI, triglyceride glucose-body mass index; TG, triglyceride; TC, total cholesterol; HDL-C, high density lipoprotein cholesterol; LDL-C, low density lipoprotein cholesterol; Apo-A1, Apolipoprotein-A1; ApoB, Apolipoprotein B; UA, uric acid; eGFR, estimated glomerular filtration rate; hs-CRP, high-sensitivity c-reactive protein; Cys C, Cystatin C; BNP, B-type natriuretic peptide.

**Table 4:** Correlation analysis of EATV and TyG-BMI with left atrial structure and function.

Variables	EATV (cm^3^)		TyG-BMI	
	*r*	*p*	*r*	*p*
LAs-s (%)	−0.230	<0.001	−0.180	0.003
LAs-e (%)	−0.187	0.002	−0.200	0.001
LAs-a (%)	−0.178	0.004	−0.071	0.249
LASI	0.218	<0.001	0.139	0.024
LAVI (mL/m^2^)	0.046	0.452	0.21	0.001

EATV, epicardial adipose tissue volume; TyG-BMI, triglyceride glucose-body mass index; LAs-s, left atrial reservoir strain; LAs-e, left atrial conduit strain; LAs-a, left atrial booster strain; LASI, left atrial stiffness index; LAVI, left atrial volume index.

### 3.4 Linear regression analysis

**Figure 5:**
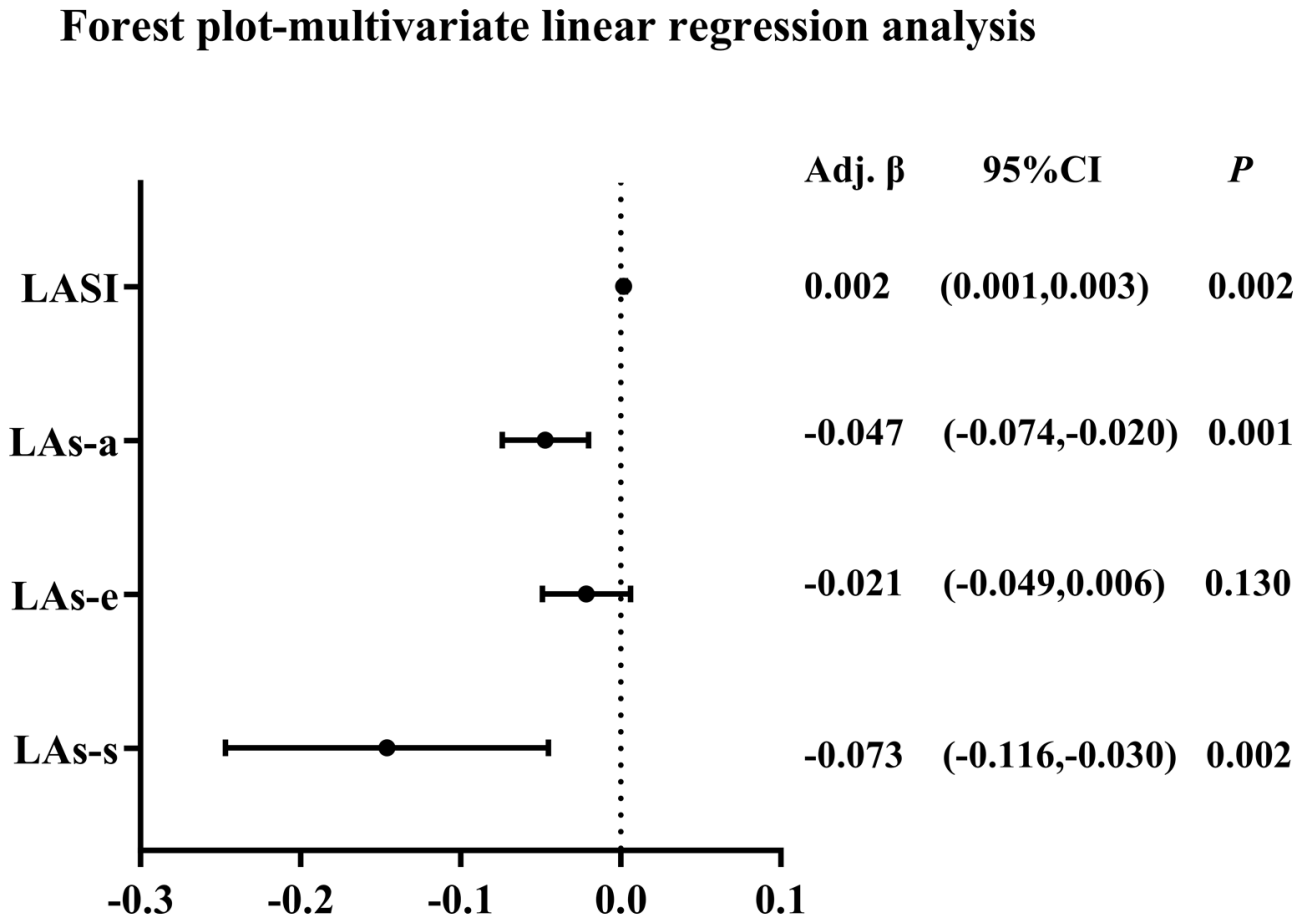
Forest plot of multivariate linear regression analysis for the association of EATV with left atrial mechanics. EATV, epicardial adipose tissue volume; LAs-s, left atrial reservoir strain; LAs-e, left atrial conduit strain; LAs-a, left atrial booster strain; LASI, left atrial stiffness index.

As shown in Table 5, Figure 5, univariate and multivariate linear regression analyses were performed to assess the independent associations between EATV and LA function. The univariate analysis revealed significant inverse associations of EATV with all LA strain parameters (LAs-s, LAs-e, LAs-a) and a positive association with LASI. After sequential adjustment for demographic factors, cardiometabolic risk factors, medication use, and LV structural and functional parameters (Models 1–4), the inverse associations with LAs-s and LAs-a, as well as the positive association with LASI, remained statistically significant. In contrast, the association between EATV and conduit strain (LAs-e) was attenuated and became non-significant following multivariable adjustment.

**Table 5:** Univariate and multivariate linear regression of EATV and left atrial structure and function.

Variables	Univariate analysis		Multivariate analysis											
			Model 1			Model 2			Model 3			Model 4		
	*β*	*p*	*β*	*p*	Adj. *R*^2^	*β*	*p*	Adj. *R*^2^	*β*	*p*	Adj. *R*^2^	*β*	*p*	Adj. *R*^2^
LAs-s	−0.092	<0.001	−0.065	0.002	0.136	−0.066	0.002	0.134	−0.069	0.001	0.131	−0.068	0.002	0.133
LAs-e	−0.048	0.001	−0.022	0.115	0.139	−0.022	0.126	0.171	−0.022	0.125	0.166	−0.021	0.130	0.175
LAs-a	−0.045	<0.001	−0.044	0.001	0.050	−0.045	0.001	0.038	−0.047	0.001	0.056	−0.047	0.001	0.061
LASI	0.002	<0.001	0.002	0.001	0.167	0.002	0.003	0.154	0.002	0.002	0.153	0.002	0.002	0.200

Beta coefficients (*β*) reported are unstandardized, adjusted *R*^2^ values are presented for each multivariate model.

Model 1, adjusted for age, sex, BMI.

Model 2, adjusted for age, sex, smoking, hypertension duration, diabetes, TyG-BMI, HDL, ApoA1, eGFR.

Model 3, adjusted for age, sex, smoking, hypertension duration, diabetes, TyG-BMI, HDL, ApoA1, eGFR, SGLT-2i, GLP-1RA, stains.

Model 4, adjusted for age, sex, smoking, hypertension duration, diabetes, TyG-BMI, HDL, ApoA1, eGFR, SGLT-2i, GLP-1RA, stains, LVEF, LVMI.

EATV epicardial adipose tissue volume, LAs-s left atrial reservoir strain, LAs-e left atrial conduit strain, LAs-a left atrial booster strain, LASI left atrial stiffness index.

Subgroup analyses were conducted to further explore these associations. When stratified by sex (Supplementary Table S1), the inverse associations of EATV with LAs-s and LAs-a remained robust and significant in males across all adjusted models. Conversely, these associations were generally attenuated and lost statistical significance in females. The positive association between EATV and LASI was significant in both sexes but appeared more pronounced in females.

When stratified by age using the median of 53 years (Supplementary Table S2), the associations were consistently stronger in the older subgroup (≥53 years). In these older patients, EATV was independently associated with worse reservoir and booster pump strain, as well as significantly higher LASI, across all multivariate models. In the younger subgroup (<53 years), these associations were largely attenuated and non-significant.

### 3.5 Mediation effect analysis

Based on our previous results showing that both higher TyG-BMI and EATV were independently associated with impaired LA function, we conducted a mediation analysis to explore whether insulin resistance, assessed by TyG-BMI, partially accounts for the association between EATV and LA mechanics.

**Figure 6:**
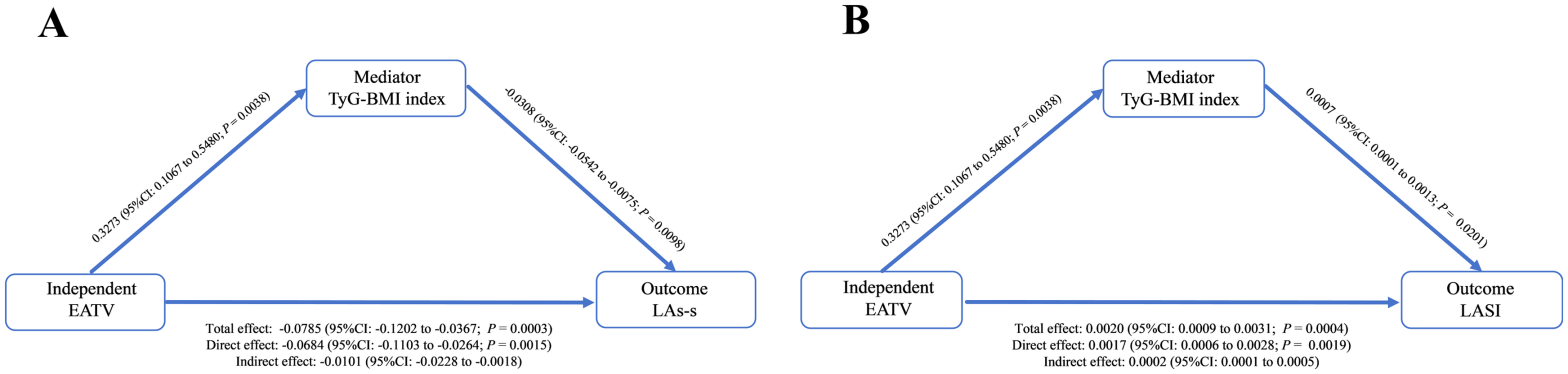
Mediation model illustrating the effect of EATV on LA reservoir strain **(A)** and stiffness index **(B)**, with TyG-BMI as a potential mediator. EATV, epicardial adipose tissue volume; TyG-BMI, triglyceride glucose-body mass index; LAs-s, left atrial reservoir strain; LASI, left atrial stiffness index.

After adjusting for age, sex, smoking, hypertension duration, diabetes, HDL-C, Apo-A1, eGFR, SGLT-2i, GLP-1RA, statins, LVEF, and LVMI, mediation analysis showed that TyG-BMI accounted for 12.87% of the association between EATV and LAs-s (indirect effect: −0.0101, 95% CI: −0.0228 to −0.0018). Furthermore, it explained 10.00% of the association between EATV and LASI (indirect effect: 0.0002, 95% CI: 0.0001–0.0005). However, no significant mediation effect of TyG-BMI was observed in the relationships between EATV and LAs-e or LAs-a (Figure 6).

## 4 Discussion

In this cross-sectional study of 264 overweight/obese hypertensive patients, we demonstrated that increased EATV was independently associated with impaired LA mechanics, as evidenced by reduced LAs-s and LAs-a, alongside elevated LASI. Crucially, these associations persisted after rigorous adjustment for cardiometabolic confounders, medications, LVEF and LVMI. Subgroup analysis further suggested that the inverse associations of EATV with LAs-s and LAs-a were stronger in males and older patients. In contrast, the positive association between EATV and LASI was consistent across sexes, but also more pronounced in older patients. This pattern indicates potential sex- and age-related differences in the link between epicardial fat and atrial dysfunction. Notably, TyG-BMI accounted for 12.87% of the association between EATV and LAs-s and 10.00% of the association between EATV and LASI. These findings allow us to propose a novel potential mechanistic hypothesis: insulin resistance may be one pathway through which EAT accumulation is associated with subclinical atrial dysfunction.

The findings of this study are consistent with prior evidence linking EAT as a critical contributor to atrial remodeling and AF pathogenesis (23). Computed tomography and magnetic resonance studies in AF patients demonstrated that greater epicardial and peri-atrial fat correlated with both structural and functional impairment of the atrium (24–27). Chen et al. (28) conducted a systematic review and meta-analysis of EAT’s impact on post-ablation AF recurrence and showed that both total and peri-atrial EATV rose in a stepwise manner from controls to paroxysmal and then persistent AF. Olsen et al. (29) reported that reduced LAs-s independently predicted subclinical atrial fibrillation, with a 4% increase in risk per 1% decrement in strain after multivariable adjustment. In a general population cohort, Cauwenberghs et al. (30) demonstrated that LAs-s < 23% was independently associated with incident AF. Similarly, Zeng et al. (31) showed that LAs-s ≤ 24.3% was independently associated with a higher risk of atrial fibrillation recurrence after catheter ablation in patients with non-valvular atrial fibrillation. Increased EATV has also been reported to correlate with HF. Emerging data have suggested that the association between EAT and HF may differ across phenotypes. In HFpEF, patients consistently exhibit higher EATV than healthy controls (32), and elevated EATV correlates with diastolic failure and adverse cardiac remodeling (33), and is independently associated with poorer outcomes in both HFpEF and HF with mid-range EF (34). In addition, Potter et al. demonstrated that LAs-s < 24% was associated with a significantly increased risk of incident asymptomatic HF (35). Moreover, Krittayaphong et al. (36) found that LAs-s < 23% is associated with a higher risk of composite cardiovascular outcomes, including death, HF, and ischemic stroke with known or suspected coronary artery disease with preserved left ventricular systolic function.

Collectively, these studies suggest that absolute LAs-s values in the range of 23–25% may serve as meaningful thresholds for identifying high-risk individuals. In our cohort, the mean LAs-s in the high EATV group was 24.4%, approaching this risk range, despite preserved left atrial volumes, indicating that subtle strain reductions may reflect early, subclinical atrial dysfunction.

This study is the first to integrate high-resolution CCTA quantification of EATV with 2D-STE derived LA strain analysis specifically in overweight/obese patients who have primary hypertension but no clinical cardiovascular disease. Our findings not only confirmed that visceral adiposity served as a marker of early atrial impairment independent of conventional risk factors, but also identified TyG-BMI, a surrogate for insulin resistance, as a potential pathway linking EAT accumulation and subclinical LA mechanical dysfunction. This targeted focus fills a critical gap, suggesting that EAT assessment could enhance risk stratification within this high-risk, preclinical hypertensive population. Our study also provided a substantive complement to prior studies. For instance, Çetin et al. (37) demonstrated that greater EAT thickness independently associated with diastolic parameters and LA dimension in hypertensive patients with preserved systolic function. However, their study did not focus on overweight/obese subjects, nor did it evaluate atrial strain mechanics. Similarly, Ng et al. (38) described associations between EATV and myocardial fat/fibrosis in a general, non-hypertensive population, underscoring metabolic connections but without addressing hypertensive remodeling.

The observed associations can be contextualized within a framework in which EAT may exert multifaceted effects on atrial myocardium through local paracrine inflammation, pro-fibrotic and electrical remodeling, and mechanistic interplay with insulin resistance. In hypertensive, obese patients, the convergence of these pathways may contribute to impair LA strain and increase stiffness, offering a coherent explanation for the associations we observed between EATV and LA dysfunction. The specific mechanisms are as follows: epicardial fat, contiguous with the atrial myocardium, secretes pro-inflammatory adipokines (Interleukin-6, Tumor Necrosis Factor-*α*, Monocyte Chemoattractant Protein-1, Angiopoietin-like protein 2) (39, 40), which diffuse into subepicardial layers to activate fibroblasts, promote extracellular matrix deposition, and trigger fibrotic remodeling (infiltrative-lipotoxic hypothesis) (41, 42); parallel mechanical restraint within the rigid pericardium elevates filling pressures and chamber stiffness (pericardial restraint hypothesis) (43). In hypertensive obese patients, these inflammatory, fibrotic, insulin resistance, and compressive forces are posited to collectively contribute to impaired LA strain and augment stiffness, as observed in our study.

Our subgroup analyses provided clinically meaningful insights. The adverse associations between EATV and impaired LA mechanics were not uniform across patient subgroups. Specifically, the inverse relationships of EATV with LA reservoir and booster pump strains were predominantly observed in males and older (≥53 years) patients. In contrast, the positive association between EATV and LASI was consistent across sexes, but also more pronounced in older patients. These findings suggest that hypertensive overweight/obese men are particularly vulnerable to EAT-mediated LA impairment, potentially due to their tendency toward visceral and epicardial fat accumulation, and that EATV predicted cardiovascular pathology preferentially in men (44–46). Sex disparities likely stem from hormonal and fat distribution differences. Estrogens are known to modulate adipose inflammation and distribution, potentially buffering women from visceral fat’s harmful paracrine effects on the myocardium. Given the limitations of subgroup analysis and the absence of formal interaction testing, the observed attenuation of associations in females should be interpreted with caution. The differences may relate to baseline sex imbalances, or distinct fat distribution pathophysiology. Consequently, the primary associations of EATV with LAs-a and LAs-s appear more robust in male participants, a finding that warrants validation in larger, balanced prospective studies to clarify the role of sex.

In addition, aging is intrinsically linked with an increase in pro-fibrotic signaling, a decline in metabolic reserve, and the accumulation of other age-related comorbidities (47, 48). In this context, the added burden of a large EATV may overwhelm compensatory mechanisms, leading to more evident impairments in LA mechanics. Our finding underscored EAT as a potent accelerant of age-related atrial myopathy in the setting of hypertension and obesity.

We note that statin use was more frequent in the high-EATV group. Beyond their lipid-lowering effects, statins exhibit pleiotropic properties, including anti-inflammatory, antioxidant, anti-thrombotic, and endothelial function-improving properties that can directly influence EAT biology and volume (49, 50). Although we adjusted for statin use, residual confounding cannot be excluded. The association may partly reflect the higher cardiovascular risk prompting statin therapy. Conversely, these very properties might attenuate the true EAT-atrial impairment, suggesting our estimates are conservative. Future studies should separate drug effects from EAT-specific pathophysiology.

Notably, the current study also examined whether insulin resistance, quantified by the TyG-BMI, plays a role in the association between EATV and left atrial dysfunction. Previous studies have revealed that epicardial fat mass is strongly associated with whole-body insulin sensitivity indices (51). Our mediation analysis confirmed that greater EATV was associated with a higher TyG-BMI, which in turn was linked to worse LA function. Specifically, this pathway accounted for 12.87% of the association between EATV and reduced LAs-s, and for 10.00% of its association with increased LASI. Collectively, this describes a pathway of associations linking EATV, TyG-BMI, and impaired LA mechanics, highlighting that epicardial adipose accumulation, through metabolic derangements, is implicated in the development of structural and functional atrial changes. This suggests the hypothesis that targeting visceral fat or insulin resistance could interfere early atrial remodeling.

## 5 Clinical implications

Our study highlights several actionable insights for clinical practice in hypertensive overweight or obese patients:

1. EATV, readily quantified by non-contrast or contrast-enhanced CT, may serve as a sensitive early imaging marker of subclinical atrial remodeling. Elevated EATV, even before overt cardiovascular disease, is associated with early impairment of atrial mechanics. Notably, the lack of association between EATV and LAVI implies that mechanical dysfunction in the context of high EATV precedes overt structural remodeling, a critical distinction for early risk stratification.
2. Refining risk stratification and tailored therapies. Incorporating EAT quantification into traditional risk frameworks could potentially refine prognostic precision. In asymptomatic or intermediate-risk individuals, adding CT-derived EATV to coronary calcium scoring has been shown to improve prediction of future cardiovascular events (52). This evidence, combined with our findings, raises the possibility that hypertensive patients with elevated EATV might benefit from more aggressive metabolic modulation, such as insulin-sensitizing agents or targeted weight-loss strategies, aimed at mitigating the adverse associations of visceral adiposity. Indeed, weight-loss interventions and newer GLP-1RA have been shown to reduce EATV in parallel with improvements in cardiac function, supporting a personalized approach guided by EAT metrics (52).
3. Integrating CCTA-Derived EAT and 2D-STE into routine care. A combined imaging pathway, leveraging routine CCTA for EATV measurement and 2D-STE for detailed atrial strain analysis, could help identify early atrial cardiomyopathy in hypertensive obese patients. Adopting a multimodal protocol in hypertension clinics would enable longitudinal monitoring of EATV and atrial mechanics, permitting timely therapeutic escalation, whether through optimized blood pressure control, anti-inflammatory strategies, or lifestyle interventions, with the goal of potentially reducing the future risk of AF or HF.

## 6 Strengths and limitations

### 6.1 Strengths

#### 6.1.1 Comprehensive imaging protocol

By integrating high-resolution CCTA volumetry of EATV with 2D-STE, we achieved precise, simultaneous assessment of both structural (EATV) and functional (LA strain) parameters. This multimodality approach overcomes the limitations of single-technique studies and enhances sensitivity for detecting subclinical atrial remodeling.

#### 6.1.2 Novel mediation analysis

Applying bootstrap-based mediation modeling allowed us to quantify the extent to which TyG-BMI mediates the relationship between EATV and LA reservoir strain and LA stiffness. This mechanistic insight elucidates a specific link between epicardial fat and LA function, strengthening the scientific rationale for viewing visceral fat as a potential target.

### 6.2 Limitations

Our study has several limitations. First, the cross-sectional, single-center design precludes causal inference. Second, residual confounding from unmeasured factors, particularly abdominal visceral fat, cannot be excluded despite multivariate adjustment. Third, measuring total rather than region-specific (e.g., peri-atrial) EATV may dilute the mechanistic specificity of our findings, as peri-atrial fat may exert more direct paracrine and mechanical effects on the adjacent atrial myocardium and be more relevant to LA mechanics. Fourth, the use of the TyG-BMI, which incorporates BMI, means its mediation effect may reflect general adiposity in addition to insulin resistance. Future prospective studies combining high-resolution CT for peri-atrial EAT quantification, direct abdominal fat imaging, and direct insulin resistance assays could help disentangle local from systemic effects on atrial dysfunction.

## 7 Conclusion

In patients with primary hypertension and overweight/obesity, increased EATV is independently linked to early left atrial remodeling, manifested by elevated LASI and reduced LAs-s and LAs-a. Mediation analysis indicated that insulin resistance was a potential mediator of these associations. Taken together, these findings highlight a potential link between epicardial adiposity, metabolic dysfunction, and early atrial mechanical abnormalities in a high-risk population. Further longitudinal studies are required to determine the temporal relationships and clinical implications of these associations.

## Data Availability

The original contributions presented in the study are included in the article/Supplementary material, further inquiries can be directed to the corresponding author.

## Supplementary material

Supplementary Table 1 Multivariable linear regression results (presenting *β* coefficients, *p*-values, and adjusted *R*²) for the association between epicardial adipose tissue volume (EATV) and left atrial strain parameters (LAs-s, LAs-e, LAs-a) and stiffness index (LASI), stratified by sex.

Supplementary Table 2 Multivariable linear regression results (presenting *β* coefficients, *p*-values, and adjusted *R*²) for the association between EATV and left atrial parameters (LAs-s, LAs-e, LAs-a, LASI), stratified by age group (cut-off: 53 years).
