# Supplementary material for: Epicardial adipose tissue volume is associated with impaired left atrial mechanics in hypertensive overweight/obese patients: the potential mediating role of insulin resistance
Source: Front Nutr. 2026 Feb 23;13:1778193. doi: 10.3389/fnut.2026.1778193 (PMC12968267; doi:10.3389/fnut.2026.1778193)
Supplement: Supplementary Table 1 — Multivariable linear regression results (presenting β coefficients, p-values, and adjusted R²) for the association between epicardial adipose tissue volume (EATV) and left atrial strain parameters (LAs-s, LAs-e, LAs-a) and stiffness index (LASI), stratified by sex. [file Table_1.DOCX]

**Table S1** Multivariate linear regression of EATV and left atrial structure and function by sex

| **Multivariate analysis** | | | | | | | | | | | | | | | | | | |
| --- | --- | --- | --- | --- | --- | --- | --- | --- | --- | --- | --- | --- | --- | --- | --- | --- | --- | --- |
|  | **Model 1** | | | **Model 2** | | | | |  | **Model 3** | | | |  | **Model 4** | | |  |
|  | **β** | ***P*** | **Adj.R^2^** | | **β** | ***P*** | **Adj.R^2^** | **β** | | | ***P*** | | **Adj.R^2^** | | **β** | ***P*** | **Adj.R^2^** |  |
| LAs-s | -0.064^a^ | 0.006 | 0.174 | | -0.061^a^ | 0.010 | 0.186 | -0.062^a^ | | | 0.010 | | 0.174 | | -0.063^a^ | 0.009 | 0.178 |  |
|  | -0.070^b^ | 0.132 | 0.136 | | -0.068^b^ | 0.151 | 0.224 | -0.062^b^ | | | | 0.188 | 0.295 | | -0.063^b^ | 0.187 | 0.277 |  |
| LAs-e | -0.022^a^ | 0.151 | 0.156 | | -0.020^a^ | 0.200 | 0.171 | -0.019^a^ | | | | 0.238 | 0.160 | | -0.019^a^ | 0.244 | 0.173 |  |
|  | -0.024^b^ | 0.463 | 0.142 | | -0.024^b^ | 0.474 | 0.231 | -0.016^b^ | | | | 0.638 | 0.271 | | -0.020^b^ | 0.564 | 0.275 |  |
| LAs-a | -0.043^a^ | 0.006 | 0.076 | | -0.042^a^ | 0.008 | 0.091 | -0.045^a^ | | | | 0.005 | 0.098 | | -0.045^a^ | 0.005 | 0.092 |  |
|  | -0.046^b^ | 0.110 | 0.022 | | -0.044^b^ | 0.143 | 0.051 | -0.046^b^ | | | | 0.123 | 0.126 | | -0.043^b^ | 0.145 | 0.126 |  |
| LASI | 0.002^a^ | 0.002 | 0.148 | | 0.001^a^ | 0.043 | 0.176 | 0.001^a^ | | | | 0.038 | 0.168 | | 0.001^a^ | 0.026 | 0.235 |  |
|  | 0.003^b^ | 0.007 | 0.315 | | 0.003^b^ | 0.009 | 0.304 | 0.003^b^ | | | | 0.015 | 0.379 | | 0.002^b^ | 0.019 | 0.370 |  |

Beta coefficients (β) reported are unstandardized, Adjusted R² values are presented for each multivariate model.

All models were sex-stratified, with suffix 'a' denoting males and suffix 'b' denoting females.

Model 1, adjusted for age, BMI;

Model 2, adjusted for age, sex, smoking, hypertension duration, diabetes, TyG-BMI, HDL, ApoA1, eGFR;

Model 3, adjusted for age, sex, smoking, hypertension duration, diabetes, TyG-BMI, HDL, ApoA1, eGFR, SGLT-2i, GLP-1RA, stains;

Model 4, adjusted for age, sex, smoking, hypertension duration, diabetes, TyG-BMI, HDL, ApoA1, eGFR, SGLT-2i, GLP-1RA, stains, LVEF, LVMI;

EATV epicardial adipose tissue volume, LAs-s left atrial reservoir strain, LAs-e left atrial conduit strain, LAs-a left atrial booster strain, LASI left atrial stiffness index.
