# Supplementary material for: Epicardial adipose tissue volume is associated with impaired left atrial mechanics in hypertensive overweight/obese patients: the potential mediating role of insulin resistance
Source: Front Nutr. 2026 Feb 23;13:1778193. doi: 10.3389/fnut.2026.1778193 (PMC12968267; doi:10.3389/fnut.2026.1778193)
Supplement: Supplementary Table 2 — Multivariable linear regression results (presenting β coefficients, p-values, and adjusted R²) for the association between EATV and left atrial parameters (LAs-s, LAs-e, LAs-a, LASI), stratified by age group (cut-off: 53 years). [file Table_2.DOCX]

**Table S2** Multivariate linear regression of EATV and left atrial structure and function by age

| **Multivariate analysis** | | | | | | | | | | | | | | | | | |
| --- | --- | --- | --- | --- | --- | --- | --- | --- | --- | --- | --- | --- | --- | --- | --- | --- | --- |
|  | **Model 1** | | | **Model 2** | | | | |  | **Model 3** | | |  | **Model 4** | | |  |
|  | **β** | ***P*** | **Adj.R^2^** | | **β** | ***P*** | **Adj.R^2^** | **β** | | | ***P*** | **Adj.R^2^** | | **β** | ***P*** | **Adj.R^2^** |  |
| LAs-s | -0.028^a^ | 0.371 | 0.129 | | -0.032^a^ | 0.328 | 0.121 | -0.032^a^ | | | 0.322 | 0.116 | | -0.035^a^ | 0.291 | 0.109 |  |
|  | -0.095^b^ | 0.001 | 0.118 | | -0.084^b^ | 0.004 | 0.118 | -0.087^b^ | | | 0.004 | 0.108 | | -0.085^b^ | 0.005 | 0.095 |  |
| LAs-e | 0.004^a^ | 0.846 | 0.164 | | 0.002^a^ | 0.912 | 0.190 | 0.002^a^ | | | 0.916 | 0.172 | | -0.002^a^ | 0.945 | 0.177 |  |
|  | -0.043^b^ | 0.021 | 0.097 | | -0.034^b^ | 0.077 | 0.122 | -0.034^b^ | | | 0.080 | 0.121 | | -0.037^b^ | 0.054 | 0.132 |  |
| LAs-a | -0.036^a^ | 0.085 | 0.021 | | -0.038^a^ | 0.080 | 0.006 | -0.039^a^ | | | 0.077 | 0.011 | | -0.038^a^ | 0.091 | 0.001 |  |
|  | -0.050^b^ | 0.007 | 0.041 | | -0.049^b^ | 0.010 | 0.026 | -0.052^b^ | | | 0.007 | 0.034 | | -0.047^b^ | 0.015 | 0.074 |  |
| LASI | 0.001^a^ | 0.694 | 0.107 | | 0.001^a^ | 0.736 | 0.102 | 0.001^a^ | | | 0740 | 0.081 | | 0.001^a^ | 0.992 | 0.106 |  |
|  | 0.003^b^ | <0.001 | 0.170 | | 0.003^b^ | <0.001 | 0.157 | 0.003^b^ | | | <0.001 | 0.153 | | 0.003^b^ | 0.001 | 0.169 |  |

Beta coefficients (β) reported are unstandardized, Adjusted R² values are presented for each multivariate model.

All models were age-stratified, with suffix 'a' denoting the younger group (<53 years) and suffix 'b' denoting the older group (≥53 years).

Model 1, adjusted for age, sex, BMI;

Model 2, adjusted for age, sex, smoking, hypertension duration, diabetes, TyG-BMI, HDL, ApoA1, eGFR;

Model 3, adjusted for age, sex, smoking, hypertension duration, diabetes, TyG-BMI, HDL, ApoA1, eGFR, SGLT-2i, GLP-1RA, stains;

Model 4, adjusted for age, sex, smoking, hypertension duration, diabetes, TyG-BMI, HDL, ApoA1, eGFR, SGLT-2i, GLP-1RA, stains, LVEF, LVMI;

EATV epicardial adipose tissue volume, LAs-s left atrial reservoir strain, LAs-e left atrial conduit strain, LAs-a left atrial booster strain, LASI left atrial stiffness index.
